# Effect of Forsythiaside A on the RLRs Signaling Pathway in the Lungs of Mice Infected with the Influenza A Virus FM1 Strain

**DOI:** 10.3390/molecules24234219

**Published:** 2019-11-20

**Authors:** Xiao Zheng, Yingjie Fu, Shan-Shan Shi, Sha Wu, Yuqi Yan, Liuyue Xu, Yiwei Wang, Zhenyou Jiang

**Affiliations:** 1 Department of Microbiology and Immunology, Basic Medicine College, Jinan University, GuangZhou 510632, China; zhengdaxiao1@163.com (X.Z.); fuyingjieanan@hotmail.com (Y.F.); shiss@jnu.edu.cn (S.-S.S.); iccy712@163.com (S.W.); Chris1202@126.com (Y.Y.); lewisxu95@gmail.com (L.X.); wangyiweiyouxiang@126.com (Y.W.); 2 Institute of Medical Microbiology, Jinan University, GuangZhou 510632, China

**Keywords:** influenza A virus FM1 mouse lung adaptive strain (H1N1; A/FM1/1/47 strain), forsythiaside A, RLRs signaling pathway, *MAVS*^−/−^ mice

## Abstract

Forsythiaside A, a phenylethanoid glycoside monomer extracted from *Forsythia suspensa*, shows anti-inflammatory, anti-infective, anti-oxidative, and antiviral pharmacological effects. The precise mechanism underlying the antiviral action of forsythiaside A is not completely clear. Therefore, in this study, we aimed to determine whether the anti-influenza action of forsythiaside A occurs via the retinoic acid-inducible gene-I–like receptors (RLRs) signaling pathway in the lung immune cells. Forsythiaside A was used to treat C57BL/6J mice and *MAVS*^−/−^ mice infected with mouse-adapted influenza A virus FM1 (H1N1, A/FM1/1/47 strain), and the physical parameters (body weight and lung index) and the expression of key factors in the RLRs/NF-κB signaling pathway were evaluated. At the same time, the level of virus replication and the ratio of Th1/Th2 and Th17/Treg of T cell subsets were measured. Compared with the untreated group, the weight loss in the forsythiaside A group in the C57BL/6J mice decreased, and the histopathological sections showed less inflammatory damage after the infection with the influenza A virus FM1 strain. The gene and protein expression of retinoic acid-inducible gene-I (RIG-I), MAVS, and NF-κB were significantly decreased in the forsythiaside A group. Flow cytometry showed that Th1/Th2 and Th17/Treg differentiated into Th2 cells and Treg cells, respectively, after treatment with forsythiaside A. In conclusion, forsythiaside A reduces the inflammatory response caused by influenza A virus FM1 strain in mouse lungs by affecting the RLRs signaling pathway in the mouse lung immune cells.

## 1. Introduction

Influenza is an acute respiratory disease, and approximately five million influenza virus infections and 250,000–640,000 deaths are annually reported worldwide [1]. Influenza viruses belong to the Orthomyxoviridae family and are classified into three serotypes: A, B, and C. The presence of segmented RNA leads to genomic and antigenic variability such as antigen conversion. Influenza A viruses have two surface proteins, hemagglutinin (HA) and neuraminidase (NA), and are further subtyped based on their antigenicity. Influenza A viruses can also obtain HA and NA from other subtypes via antigen conversion. Such fragment recombination events can occur in cells infected with different human and animal influenza viruses, and the resulting viruses can encode for completely novel antigenic proteins. An influenza pandemic can occur if the population is susceptible to and cannot elicit an immune response against the new viruses produced by antigen-switching mechanisms. 

Influenza virus infection is a persistent global health threat associated with high morbidity and mortality. Current antiviral drugs include M2 ion channel inhibitors and neuraminidase inhibitors. The Food and Drug Administration of the United States has approved four antiviral drugs, namely amantadine, rimantadine, zanamivir, and oseltamivir for the treatment of influenza, but due to the rapid mutations in the target virus protein, the use of amantadine as an M2 inhibitor is limited [2]. The neuraminidase inhibitors, zanamivir and oseltamivir, are effective against influenza A and B viruses and cause fewer adverse effects. However, the main concern with their use is the emergence of resistance [3]. The efficacy of existing antiviral drugs is limited by the emergence of resistant strains and recombinant virulent strains, thus highlighting the urgent need for alternative strategies to overcome pandemic influenza [4].

**Figure 1:**
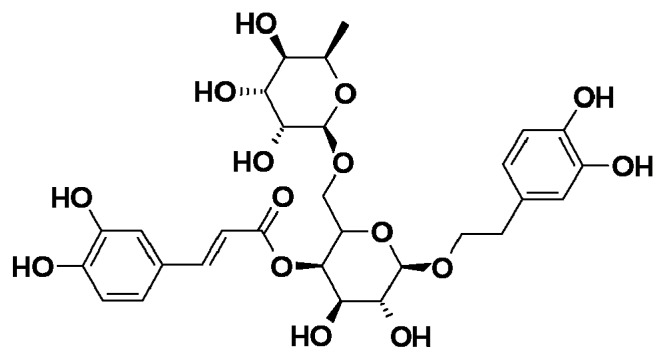
Chemical structure of forsythiaside A.

We previously reported the therapeutic effects of some traditional Chinese medicine prescriptions in mice infected with influenza virus in different environments [5,6]. Compounds used in traditional Chinese medicine contain several different drugs, and the precise components that have anti-influenza action remain unidentified. However, traditional Chinese medicine monomers, including berberine and baicalin, among others, have been reported to be effective for the treatment of influenza [7,8]. *Forsythia suspensa* is commonly used in traditional Chinese herbal medicine to clear away heat and detoxify toxins and forsythiaside A (FTA) is one of the main active ingredients of *Forsythia suspensa*. FTA is a phenylethanoid glycoside with anti-inflammatory, anti-infective, anti-oxidant, and other pharmacological properties. In addition, FTA acts as an immunomodulatory agent by regulating different types of cytokines and is considered a potential therapeutic agent [9,10]. In fact, FTA was reported to show a significant therapeutic effect against influenza A virus infection in mice [11]. The molecular structure is shown in Figure 1. 

Mammalian cells have the intrinsic capacity to detect viral pathogens and to initiate an antiviral response that is characterized by the induction of interferons (IFNs) and proinflammatory cytokines. A delicate regulation of the signaling pathways that lead to cytokine production is needed to ensure effective clearance of the virus, while preventing tissue damage caused by excessive cytokine release [12].

Innate immunity is the first line of defense against invading pathogens. Rapid and efficient detection of pathogen-associated molecular patterns via pattern-recognition receptors (PRRs) is essential for the host to mount defensive and protective responses. The innate antiviral immune response begins with the detection of evolutionarily conserved structures by a group of strain-coded PRRs, known as pathogen-associated molecular patterns (PAMPs). According to their cell localization, ligand specificity, and function, PRRs are classified into different families, including Toll-like receptors, nucleotide-binding oligomerization domain-like receptors, C-type lectin receptors, and retinoic acid-inducible gene-I (RIG-I)-like receptors (RLRs) [13,14,15]. RIG-I is critical to trigger anti-viral and inflammatory responses and control viral replication in response to cytoplasmic virus-specific RNA structures. Further, it is involved in the identification of multiple RNA viruses such as influenza A virus, Japanese meningitis virus, and paramyxovirus in the cytoplasm [16,17]. Activated RIG-I signals interact with the adapter protein MAVS leading to a signaling cascade that activates the transcription factors IRF3 and NF-κB. These actions induce the expression of antiviral gene products and the production of type I and III interferons that lead to an antiviral state in the infected cell and surrounding tissue [18].

In this study, *MAVS*^−/−^ mice were compared with C57BL/6J mice to evaluate key factors in the RLRs signaling pathway after influenza infection. The use of *MAVS*^−/−^ mice provided an excellent opportunity to further verify the regulatory effect of FTA on the RLRs signaling pathway following influenza A virus infection.

## 2. Results

### 2.1. Changes in Body Weight

**Figure 2:**
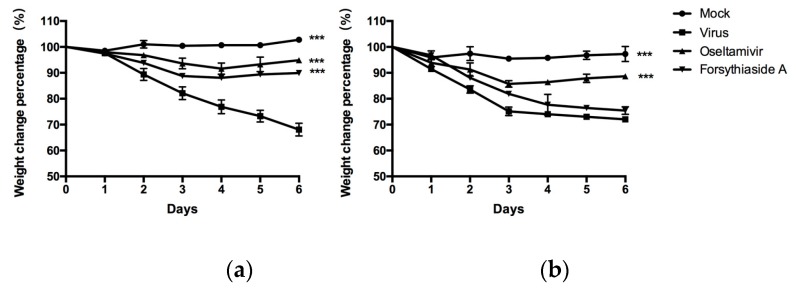
Effects of forsythiaside A (FTA) on weight loss in mice infected with influenza virus. (**a**) and (**b**) indicate the weight loss in normal wild type mice and *MAVS*^−/−^ mice (*n* = 6). Statistical analysis was performed by ANOVA with repeated measures. * *p* < 0.05, ** *p* < 0.01, *** *p* < 0.001.

After influenza virus infection, the survival and body weight of mice were monitored daily. Both C57BL/6J and *MAVS*^−/−^ mice from the virus-infected groups showed decreased activity and delayed response after the second day of viral infection; no such changes were observed in the normal control group. After the third day, the C57BL/6J and *MAVS*^−/−^ mice in the virus control group showed shaggy hair, anorexia, and rapid weight loss. However, the flu symptoms in the oseltamivir and FTA groups improved significantly and the weight loss decreased (*p* < 0.05). The weight of mice in the normal control group increased slightly or remained unchanged, whereas that of mice in the virus control group decreased rapidly (Figure 2). Compared with the virus group, the weight of oseltamivir and FTA groups in C57BL/6J mice was significantly different (*p* < 0.05). No significant difference was observed between the virus control and FTA groups of *MAVS*^−/−^ mice (*p* > 0.05).

### 2.2. Lung Index

**Figure 3:**
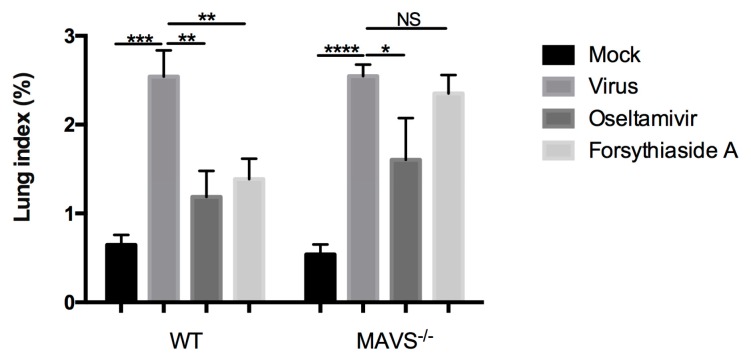
Effects of forsythiaside A on lung index of influenza virus-infected mice. At day 6 post infection, the mice were sacrificed, and the lungs were completely resected. Lung index = lung weight/body weight × 100%. (*n* = 6) * *p* < 0.05, ** *p* < 0.01, *** *p* < 0.001, NS, *p* > 0.05.

Next, we determined the lung index, which is considered indicative of the severity of pulmonary inflammation. Since inflammatory exudates may increase lung weight, the higher the lung index, the more severe the pulmonary inflammation. The lung index for the viral control groups was significantly higher than that of the mock-treated controls of C57BL/6J mice and *MAVS*^−/−^ mice, indicating the occurrence of an inflammatory response in the mouse lungs. However, in C57BL/6 mice, the lung index of the oseltamivir and FTA groups was significantly lower than that in the virus control group. There was no significant difference between the lung indices of the different *MAVS*^−/−^ mouse groups (*p* > 0.05). These results indicate that FTA significantly reduced the lung index of C57BL/6J mice but not that of *MAVS*^−/−^ mice (Figure 3).

### 2.3. Histopathological Analyses in the Lung Tissue

**Figure 4:**
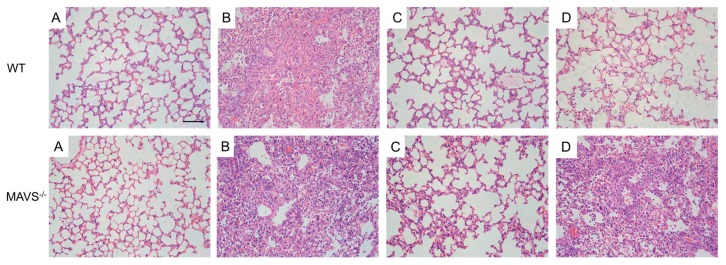
Lung histopathology of WT and *MAVS*^−/−^ mice infected with influenza virus. At day 6 post infection, the lungs were collected and sectioned for histopathological analysis. (**A**–**D**) indicates the mock-treated controls, virus group, oseltamivir group, and forsythiaside A group (*n* = 6). Bars = 100 μm.

Further, the lung tissues were harvested and analyzed morphologically. In the normal control C57BL/6J and *MAVS*^−/−^ mice, the alveolar structure was intact, and the morphology was regular; the epithelium and muscular layers of the bronchial mucosa were intact, and there was no infiltration of inflammatory cells around them. The histopathological sections of the lungs from influenza virus-infected mice showed diffuse lesions in the alveoli, alveolar septum, and bronchi, and showed a high number of lymphocytes infiltrated in the interstitial lung tissues. Diffuse damage in alveoli, alveolar septum, and bronchus and increased lymphocyte infiltration in the pulmonary interstitium were observed in the virus control group, compared to the normal control group of C57BL/6J and *MAVS*^−/−^ mice. In C57BL/6J mice, compared with the virus control group, the oseltamivir and FTA groups showed a significant reduction in inflammatory cell infiltration in the lung tissue and showed a relatively complete bronchial mucosa. However, in *MAVS*^−/−^ mice, the FTA group showed marked vasodilation and hyperemia, alveolar dilatation and even rupture, pulmonary interstitial edema, and infiltration of inflammatory cells in alveoli. Thus, FTA can significantly reduce lung inflammation in C57BL/6J mice but not in *MAVS*^−/−^ mice, indicating the involvement of the RLRs signaling pathway in the anti-influenza role of FTA (Figure 4).

### 2.4. Amplification of Influenza A Virus in Mouse Lungs

**Figure 5:**
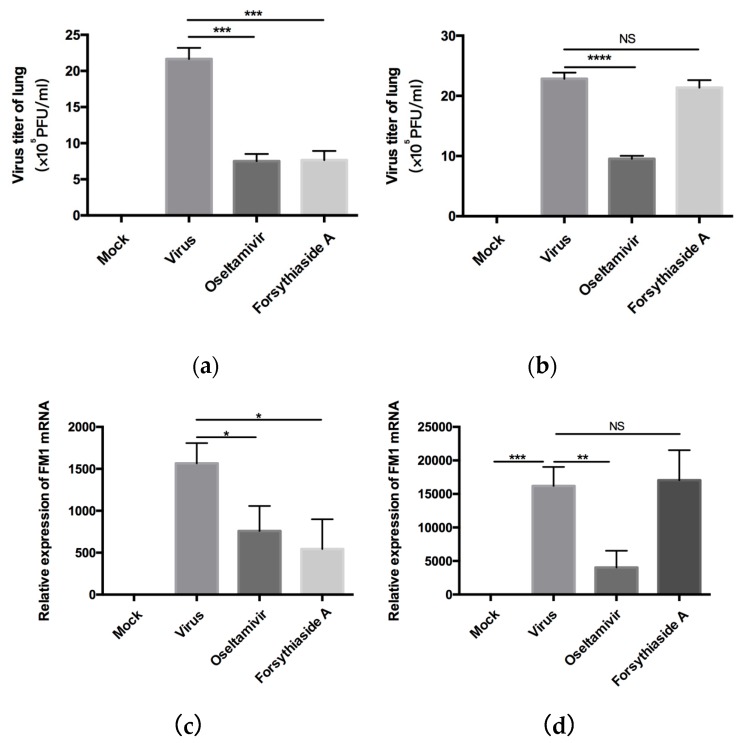
(**a**) and (**b**) indicate the influenza A virus FM1 viral titer in the lung tissues of WT and *MAVS*^−/−^ mice at day 6 post infection (*n* = 6). (**c**) and (**d**) indicate expression of the influenza A virus FM1 mRNA in the lung tissues of WT and *MAVS*^−/−^ mice at day 6 post infection (*n* = 6). * *p* < 0.05, ** *p* < 0.01, *** *p* < 0.001, NS, *p* > 0.05.

Next, the results of plaque formation experiment showed replication of the virus in mice. In C57BL/6J mice, the viral titer of the virus group was significantly higher than that of the drug group, while in *MAVS*^−/−^ mice, the viral titer of the forsythiaside A group was not significantly different from that of the virus group. Meanwhile, we used RT-qPCR to investigate the expression of viral mRNA in mouse lungs. For both C57BL/6J and *MAVS*^−/−^ mice, the expression of FM1 mRNA was not detected in the lungs from the normal control group but was remarkably high in those from the virus control group. For both C57BL/6J and *MAVS*^−/−^ mice, the expression of FM1 mRNA in the oseltamivir group was lower than that in the virus control group. However, the expression of FM1 mRNA in the FTA group of C57BL/6J mice was significantly lower than that in the virus control group; such a decrease was not observed in the *MAVS*^−/−^ mice. These results show that FTA led to decreased expression of viral FM1 mRNA in the lungs of mice infected with influenza A virus and could prevent virus replication by acting via the inflammatory response to the RLRs signaling pathway (Figure 5).

### 2.5. Relative mRNA Expression of RIG-I, MAVS, and NF-κB

**Figure 6:**
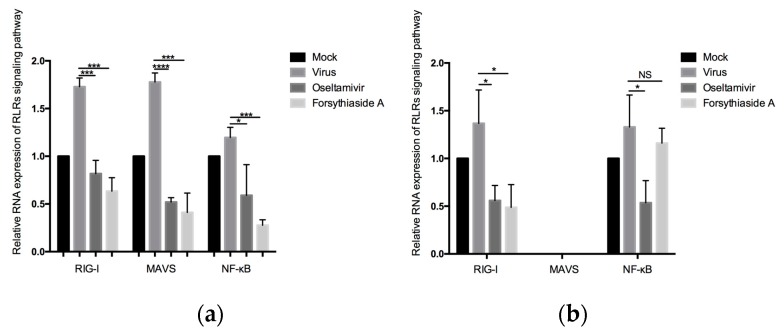
Relative RNA expression of retinoic acid-inducible gene-I–like receptors (RLRs) signaling pathway. (**a**) and (**b**) indicate the relative expression of retinoic acid-inducible gene-I (RIG-I), *MAVS,* and NF-κB in RLRs signaling pathways of WT and *MAVS*^−/−^ mice at day 6 post infection (*n* = 6). * *p* < 0.05, ** *p* < 0.01, *** *p* < 0.001, NS, *p* > 0.05.

The RLRs signaling pathways are well-known innate immune pathways that recognize influenza viruses in the cytoplasm and regulate the corresponding immune responses. Therefore, to study the effect of FTA on the RLRs signaling pathway during a viral infection, we measured the relative expression of RIG-I, MAVS, and NF-κB in the mouse lung tissues. The relative expression of RIG-I, *MAVS*, and NF-κB mRNA in the viral control groups was higher than that in the mock-treated controls of C57BL/6J and *MAVS*^−/−^ mice (*p* < 0.05). In C57BL/6J mice, the relative expression of RIG-I, MAVS, and NF-κB mRNA in the oseltamivir group and FTA group were significantly lower than that in the virus control group. In *MAVS*^−/−^ mice, the relative expression of RIG-I mRNA in the oseltamivir group and FTA group was significantly lower than that in the virus control group, whereas the expression of the downstream gene NF-κB did not decrease significantly. Thus, FTA downregulated the expression of genes involved in the RLRs signaling pathway (Figure 6).

### 2.6. Protein Expression of RIG-I, MAVS, and NF-κB

**Figure 7:**
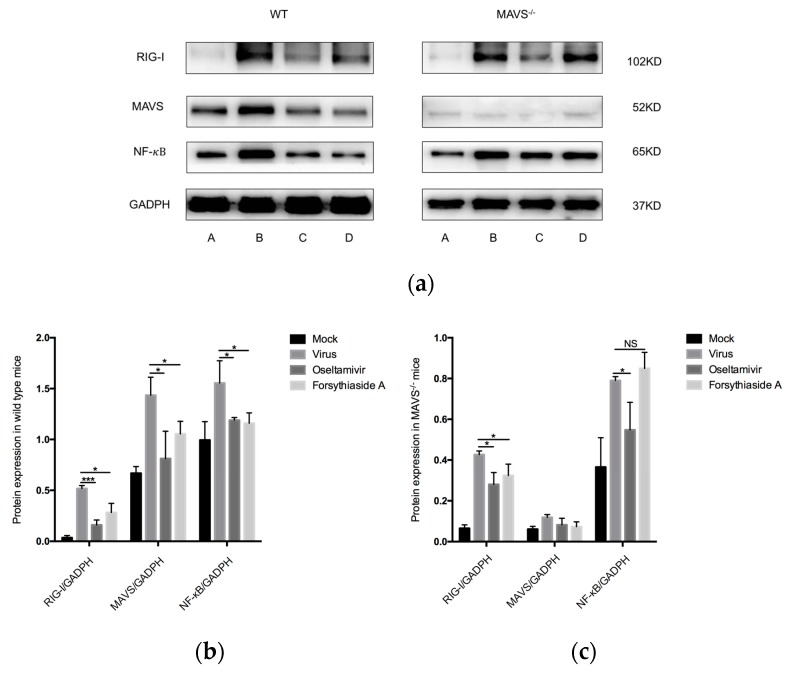
Western blot analysis of the expression of related proteins in the RLRs signaling pathway at day 6 post infection. (**a**) indicates the mock-treated controls, virus group, oseltamivir group, and forsythiaside A group. (**b**) and (**c**) represent protein expression levels of RIG-I, MAVS, and NF-κB in wild WT and *MAVS*^−/−^ mice, respectively (*n* = 6). * *p* < 0.05, ** *p* < 0.01, *** *p* < 0.001, NS, *p* > 0.05.

To further investigate the effect of FTA on the innate immune response after influenza virus infection, we used Western blotting to determine the protein levels of RIG-I, MAVS, and NF-κB in the lung tissue. In C57BL/6J mice, the levels of RIG-I, MAVS, and NF-κB in the virus control group were significantly higher than those in mock-treated controls, but those in the oseltamivir and FTA groups were significantly lower than those in the virus control group. In *MAVS*^−/−^ mice, the levels of RIG-I and NF-κB in the virus control group were significantly higher than those in the mock-treated controls, and the levels of RIG-I in the oseltamivir and FTA groups were significantly higher than those in the virus control group. However, the level of NF-κB in the oseltamivir group was significantly lower than that in the virus control group; FTA had no effect on the expression of NF-κB protein in *MAVS*^−/−^ mice. These results indicate that FTA downregulated the levels of the proteins involved in the RLRs signaling pathway (Figure 7). 

### 2.7. Classification of CD4+ T Lymphocytes

**Figure 8:**
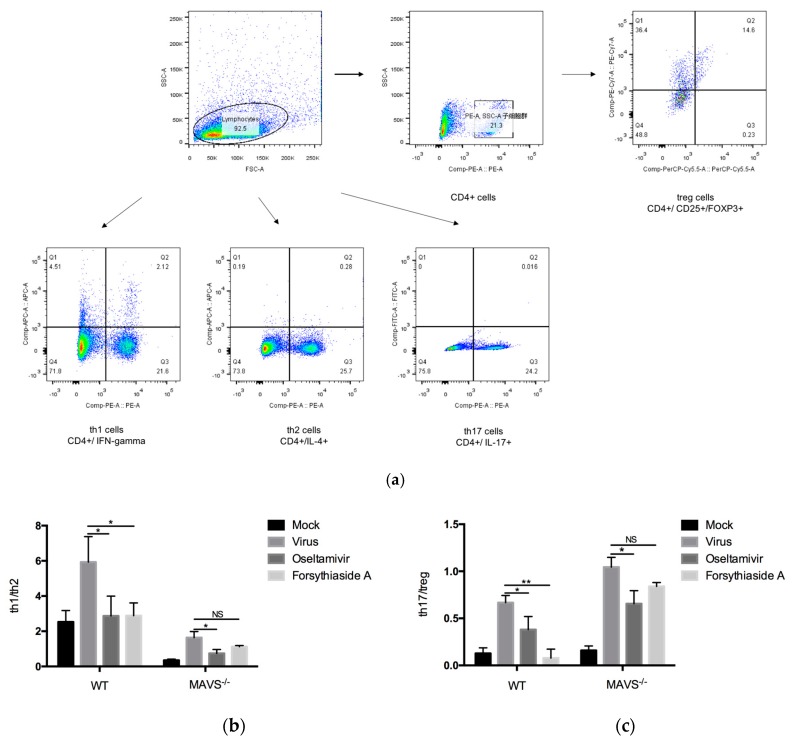
Flow cytometry for detection of Th1/Th2 and Th17/Treg in CD4+ T cell subsets at day 6 post infection. (**a**) represents flow cytometry analysis of Th1, Th2, Th17, and Treg cell subsets. (**b**) and (**c**) represent the ratio of Th1/Th2 and Th17/Treg cell subsets (*n* = 6). * *p* < 0.05, ** *p* < 0.01, *** *p* < 0.001, NS, *p* > 0.05.

The balance of Th1/Th2 and Th17/Treg is important for maintaining immune function. Therefore, to further verify the inflammatory response in mice, we used flow cytometry to determine the profile of the T cell subsets, mainly Th1, Th2, Th17, and Treg cells, in the spleen tissues of mice. In C57BL/6J mice, compared with the mock-treated controls, the virus control group showed increased Th1/Th2 and Th17/Treg ratios; compared with the virus control group, the oseltamivir and FTA groups showed decreased Th1/Th2 and Th17/Treg ratios. In *MAVS*^−/−^ mice, a significant decrease in the Th1/Th2 and Th17/Treg ratios was observed only in the oseltamivir group, compared to the virus control group (Figure 8).

## 3. Discussion

Influenza virus infection is an important public health concern. In humans, influenza viruses cause mild to severe respiratory infections, which can be fatal in severe cases. When the influenza virus infects the respiratory cells, both specific and non-specific immune responses are stimulated [19]. In the early stage of infection, a large number of viruses stimulate the epithelial cells, monocytes, and phagocytes to produce several cytokines, chemokines, inflammatory factors, and other antiviral proteins, which trigger exudation in the respiratory epithelial cells and the subsequent production of a large quantity of exudates [20]. RLRs are important pathogen-recognition receptors that recognize RNA viral infections. The RLRs signaling pathway mainly comprises a protein containing the caspase recruitment domain (CARD) located on the mitochondrial membrane and the mitochondrial antiviral signaling protein (MAVS) [21]. RLRs signaling pathway relies on the assembly of the MAVS (also known as IFN-β promoter stimulator; IPS-1) signalosome, which drives downstream activation of transcriptional response, induces interferon, antiviral and immunoregulatory genes, controls viral replication and transmission, and regulates adaptive immune responses [22]. The activation of RLRs signaling via such ligand interactions helps to initiate an immune response to viral infection. The expression of RIG-I, MAVS, and NF-κB p65 was reported to increase significantly after infection with influenza virus, suggesting that the RLRs signaling pathway and its downstream signaling factors are activated in immune cells [23]. 

In vitro and in vivo experiments have shown the effect of FTA against the influenza virus [11,24]. In this study, we investigated the effect of FTA treatment on the RLRs signaling pathway in the lungs of C57BL/6J and *MAVS*^−/−^ mice infected with influenza A virus FM1 strain. We validated the antiviral activity of FTA by establishing a mouse model for influenza virus. Currently, the prevalent influenza virus is sensitive to oseltamivir, and only a few sporadic cases of drug resistance have been reported. Hence, the current influenza treatment is strongly dependent on neuraminidase inhibitors, especially oseltamivir [25,26]. Therefore, in this experimental design, mice treated with oseltamivir were used as the positive drug controls.

First, we detected the virus titer of influenza virus in the lungs of mice by a plaque formation experiment after C57BL/6J mice were infected with influenza A virus FM1 strain. The virus titer of forsythiaside A group was significantly lower than that of the virus control group. We determined the levels of influenza A virus FM1 mRNA in the lung tissues of mice. These levels were significantly higher in the virus control group than in the other groups; the levels in the oseltamivir and FTA groups were similar to the C57BL/6J mice. The body weight and the lung index of the FTA and the positive drug group were not significantly different. Moreover, the alveolar structure of the mouse lung and the degree of inflammatory cell infiltration were further alleviated in the FTA group, compared to the positive drug control. This suggests that FTA can control the inflammation caused by influenza A virus infection. Second, we used *MAVS*^−/−^ mice to investigate whether the antiviral role of FTA involves the RLRs signaling pathway in the lungs. FTA treatment was not found to be effective in the *MAVS*^−/−^ mice infected with the influenza A virus FM1 strain.

RT-qPCR and Western blotting were used to investigate the downregulation of gene and protein expression of factors involved in the RLRs signaling pathway. The results suggest that FTA functions as an anti-influenza agent by downregulating RIG-I, MAVS, and NF-κB p65, the key factors of the RLRs signaling pathway. In our experimental results, it was found that although the *MAVS* gene was knocked out in *MAVS*^−/−^ mice, there was still a small amount of expression of RIG-I. The reason for this situation may be due to the complexity of the regulatory mechanism of the in vivo signaling pathway after influenza virus infection, and it is related to the role of various natural immune pathways in simultaneously exerting anti-influenza virus activity. At the same time, the literature has demonstrated that the production of IFN-I through the TLR7 signaling pathway after influenza virus infection plays a role in immune regulation [27]. In addition, studies have shown that resting cells express low levels of RIG-I, and natural RNA extracted from virus-infected cells, especially viral RNA genome or viral replication intermediates, have been shown to activate RIG-I when stimulated by interferon or after viral infection [28]. In conclusion, the possible mechanism of RIG-I expression in *MAVS*^−/−^ mice will be further determined in future studies. In addition, NF-κB is activated by multiple families of viruses, including human immunodeficiency virus-1 (HIV-1), human t-lymphotropic virus-I (HTLV-1), hepatitis B virus (HBV), hepatitis C virus (HCV), epstein-barr virus (EBV), and influenza virus. This activation may serve several functions: to promote viral replication, prevent virus-induced apoptosis, and mediate the immune response to the invading pathogen [29,30]. In fact, NF-κB promotes viral replication early in the viral life cycle, but the host immune system recognizes viral antigens after viral proliferation. Therefore, the viral immunity accounts for the dynamic balance between activation and inhibition of NF-κB [31]. FTA may inhibit the activation of NF-κB in the innate immune signaling pathway, thereby inhibiting the production of excessive inflammatory responses.

CD4+ T cells are necessary for protective immune response after virus infection. After virus infection, immature CD4+ T cells proliferate and produce heterogeneous cell subsets, which play unique roles in the immune response. CD4+ T cells can differentiate into Th1, Th2, Th17, Th22, T follicular helper cells, or Treg cells [32]. Th1 and Th2 cells mediate the differentiation of each other, inhibit each other, and are in equilibrium. An imbalance in the Th1/Th2 ratio often causes various diseases. The differentiation of Treg and Th17 cells is mutually inhibited and negatively regulated, and TGF-β plays an important role in this balance. Normally, TGF-β induces the initial CD4+ T cells to differentiate into Treg cells. When accompanied by infection or inflammation, IL-6, and TGF-β initiate the differentiation of the initial CD4+ T cells into Th17 cells, thereby inducing chronic inflammatory response dominated by Th17 cells. Therefore, they can regulate the balance of inflammation. IL-17 can effectively mediate the proinflammatory response in the early stage of infection or injury. Treg cells play an important role in preventing the occurrence of autoimmune diseases and controlling gastrointestinal inflammation [33,34].

In this study, flow cytometry was used to detect the balance between Th1/Th2 and Th17/Treg. In C57BL/6J mice, the ratio of Th1/Th2 and Th17/Treg in the viral control group was significantly higher than that in the mock-treated controls, indicating that the infection with the influenza A virus FM1 strain could promote the differentiation of CD4+ T cells into Th1 and Th17 cells. These results demonstrate that the infection with the influenza A virus FM1 strain can activate the immune system, increase the ratio of Th1/Th2 and Th17/Treg, and enhance the pro-inflammatory response. Furthermore, oseltamivir is a neuraminidase inhibitor, which can interfere with the release of progeny influenza virions from the surface of infected host cells. In doing so, the neuraminidase inhibitors prevent virus infection of new host cells and thereby halt the spread of infection in the respiratory tract [35]. The neuraminidase might also have a role early in influenza infection of the human airway epithelium [36]. Excessive inflammatory storm will not occur, so oseltamivir is able to reduce the proportion of Th1/Th2 and Th17/Treg after virus infection. FTA treatment enhanced the immunoregulatory function, alleviated the inflammatory response, and reduced the immunopathological damage in the lungs of influenza virus-infected mice. The possible reason is that FTA reduces virus production, while T cell response is not activated. On the other hand, the decrease of pro-inflammatory cytokines and the increase of anti-inflammatory cytokines after FTA treatment in turn inhibit the activation of T cell immune response.

In short, we demonstrated that FTA can control inflammation caused by influenza A virus infection and improve the prognosis of infection via downregulation of the RLRs signaling pathway. Further understanding of the precise molecular mechanisms underlying these processes can provide new strategies for antiviral therapy. The RLRs pathways can serve as targets to control viral infection and develop immunosuppressive strategies to control inflammation or specific autoimmune diseases.

## 4. Materials and Methods

### 4.1. Animals

Healthy, 6–8-week-old female C57BL/6J mice (Guangdong Laboratory Animal Center, Guangdong, China) and B6.129-Mavs<tm1zjc>/J mice (The Jackson Laboratory, Sacramento, CA, USA) were housed under specific-pathogen-free conditions, respectively abbreviated as WT and *MAVS*^−/−^. All knockout mice were backcrossed for more than 10 generations to C57BL/6J mice. All the mice were domesticated in an animal biosafety level 2 (ABSL-2) container at least 3 days before the experiments. All animal experiments were approved by and conducted under the supervision and evaluation of the Experimental Animal Ethics Committee of Jinan University (Approval NO. IACUC-20191120-06) (Guangdong Province, China).

### 4.2. Grouping

Twenty-four C57BL/6J and 24 *MAVS*^−/−^ mice were randomly divided into four groups (*n* = 6) as follows: mock-treated control group, virus control group, oseltamivir group, and FTA group. The mouse groups were housed separately.

### 4.3. Virus

The mouse lung-adapted strain of influenza A virus FM1 (H1N1, A/FM1/1/47 strain) frozen in liquid nitrogen was used for infecting the mice [37]. The virus was provided by the Department of Microbiology and Immunology, Basic Medical College of Jinan University, Guangzhou, Guangdong Province, China. The influenza A virus FM1 strain was used after chicken embryo amplification and each chicken embryo allantoic fluid was harvested. All chicken embryo allantoic fluid was mixed and immediately packed in Eppendorf tubes, each of which was 2 mL, titrated, and stored in liquid nitrogen. The virus infection experiments were performed in the ABSL-2 laboratory of the Department of Microbiology and Immunology, Jinan University, Guangzhou, Guangdong Province.

### 4.4. Drugs

Oseltamivir phosphate capsules were obtained from Yichang Yangtze River East Sunshine Pharmaceutical Ltd. (Lot H20065415). FTA standard (product number 111810-201707) was purchased from the National Institute for the Control of Pharmaceutical and Biological Products (Beijing, China). The purity of FTA was 97.2%.

### 4.5. Influenza Mouse Models

Mice from each group were marked and weighed. The mice were anesthetized by intraperitoneal injection of 6% chloral hydrate solution. Mice from the virus control, oseltamivir, and FTA groups were infected with 50 μL of 50% LD50FM1 virus by nasal drip; normal control mice were administered the same amount of saline via nasal drip. After infection, the mice were returned to their individual ventilated cages for normal feeding. The general survival status, body weight, hair, and other parameters were observed and recorded daily.

### 4.6. Antiviral Drug Treatment

At 24 h after infection with the influenza A virus FM1 strain, the oseltamivir and FTA groups were administered at the optimal dose in mice. The mice in the oseltamivir and FTA groups were intragastrically administered 30 mg/kg (0.4 mL/d per mouse) oseltamivir and 20 mg/kg (0.4 mL/d per mouse) FTA, respectively, for 5 days. Both oseltamivir and FTA were mixed in normal saline and then administered. The mock-treated control and virus control group mice were intragastrically administered 0.4 mL/d normal saline per mouse for 5 days. Mice were observed daily, and the survival, body weight, and symptoms were monitored. The mice were euthanized and dissected at day 6 post infection, and the lungs, spleen, and other tissues were harvested. Three mice in each group were randomly selected and divided into two parts. RT-q PCR and Western blot were used to detect the expression of key factors. The lung tissues of other mice were selected for histopathological sections.

### 4.7. Virus Titer Determination

Plaque forming unit (PFU/mL) = (number of plaques per pore/inoculation volume per pore) × virus dilution. The lungs of mice were harvested on the 6th day after infection and homogenized in 1 mL Dulbecco’s modified Eagle medium. Plaque assays on MDCK cells were used to determine viral titers in the supernatant. MDCK cells were inoculated on the cell culture plate, and the supernatant of lung tissue was diluted ten times continuously after the cells were fully grown. The holes were washed with PBS and diluted with lung homogenate. Then the cell culture plate was moved to 37 °C for 1 h. The mixture of 2% carboxymethylcellulose (CMC) and culture medium was covered and cultured for 4 days. Subsequently, 4% paraformaldehyde was added for 1 h. Sufficient crystal violet was added to the well to cover the bottom, stained for 15 min, and then slowly rinsed with distilled water to remove the crystal violet solution, and the results were observed.

### 4.8. Weight Change

The body weights of the mice in each group were recorded daily, and the body weight changes were determined. The percentage of weight change was calculated using the following formula: percentage of weight change = (body weight on day N/body weight on day 0) × 100%.

### 4.9. Lung Index

The pulmonary tissues harvested from mice were rinsed twice using normal saline and dissected. The surface water was absorbed using a filter paper, and the lung weight was recorded. The lung index was calculated using the following formula: lung index = (lung weight/body weight) × 100%.

### 4.10. Histopathological Examination

The harvested lung tissues were fixed with 4% polyformaldehyde, embedded in paraffin, and sliced into 5-μm-thick sections. The tissue samples were dewaxed, hydrated, stained with hematoxylin and eosin, and dehydrated. Finally, the histopathological conditions were observed and photographed under the microscope.

### 4.11. Quantitative Reverse Transcription Polymerase Chain Reaction (RT-qPCR)

The mRNA expression of RIG-I, MAVS, NF-κB p65, and the relative replication of influenza A virus in the lung were detected by RT-qPCR. RNAiso plus (TaKaRa, Kusatsu, Japan) was used to extract total RNA from homogenized lung tissue. After detecting the quality of the RNA, cDNA synthesis and real-time PCR were performed using the Prime Script RT kit and SYBR Green PCR Master Mix (TaKaRa, Kusatsu, Japan). The primers for GADPH, RIG-I, MAVS, NF-*κ*B p65, and FM1 were designed and synthesized using the corresponding cDNA as the templates. The designed primers were PCR amplified to detect the amplification efficiency and product specificity. The primer sequences (Shanghai Generay Biotech Co. Ltd., Shanghai, China) are listed in Table 1. CFX Connect Real Time PCR Detection System (Bio-Rad, Berkeley, CA, USA) was used to detect the gene expression. qPCR protocol was: 95 °C, 30 s; 95 °C, 5 s; 60 °C, 30 s; with 40 amplification cycle; 95 °C, 10 s. Each sample was loaded in triplicate and the experiment was performed three times. Finally, the relative expression of the gene was calculated by the cyclic threshold (2^−ΔΔCt^) method [38]; GADPH was used as the internal reference.

### 4.12. Western Blot for RIG-I, MAVS, and NF-κB p65

Total protein was extracted from the lung tissue homogenate after RIPA lysis buffer addition (Multi Sciences, Hangzhou, China). Proteins were quantified using the BCA assay (Multi Sciences, Hangzhou, China) method. The proteins were denatured using a water bath at 100 °C for 5–10 min. The denatured proteins were isolated using 10% sodium dodecyl sulfate-polyacrylamide gel electrophoresis (SDS-PAGE) and transferred onto a polyvinylidene fluoride (PVDF) membrane (Millipore, Darmstadt, Germany). The membrane was blocked with 5% skim milk powder for 90 min and washed with Tris-Buffered Saline with Tween 20 (TBST) three times (10 min each time). Then, the membranes were incubated with primary rabbit anti-mouse monoclonal antibodies against RIG-I (CST, 4200S, 1:1000), MAVS (CST, 4983S, 1:1000), NF-κB p65 (CST, 8242S, 1:1000), and GADPH (CST, 2118S, 1:1000) at 4 °C. Then, the membranes were incubated with secondary antibody (horseradish peroxidase-conjugated goat anti-rabbit IgG; 1:5000; Multi Sciences, Hangzhou, China). Protein bands were detected using an electrochemiluminescence kit (Multi Sciences, Hangzhou, China), according to the manufacturer’s instructions and analyzed using ImageJ software.

### 4.13. Flow Cytometry

The mouse spleen tissue obtained from each mouse was washed with serum-free 1640 medium and homogenized. The supernatants were collected and lymphocytes were separated using lymphocyte separation liquid (Multi Sciences, Hangzhou, China) to obtain peripheral blood mononuclear cells (PBMCs), according to the manufacturer’s instructions and adjusted to a concentration of 1 × 10^6^ cells/mL. Different T cell subsets, mainly Th1, Th2, Th17, and T regulatory (Treg) cells were detected by flow cytometry using antibodies against CD4, CD25, FOXP3, IFN-gamma, IL-4, and IL-17 (Table 2). Flow cytometry was performed using the FACS Verse flow cytometer (Becton Dickinson Biosciences, Franklin Lakes, NJ, USA) and analyzed using FlowJo v10 (FlowJo, Ashland, OR, USA).

### 4.14. Statistical Analysis

Statistical analysis was performed using the GraphPad Prism software 6.0. The results were expressed as mean ± standard deviation (SD). Analysis was performed using ANOVA with repeated measures and independent sample *t*-test; *p* < 0.05 was considered to indicate statistical significance.

## 5. Conclusions

Forsythiaside A reduces the inflammatory response caused by influenza A virus FM1 strain in mouse lungs by affecting the RLRs signaling pathway in the mouse lung immune cells.

## Figures and Tables

**Table 1:** Primers used for quantitative reverse transcription polymerase chain reaction (RT-qPCR) analysis.

Gene	Primer (5′-> 3′)	Sequence (5′-> 3′)
RIG-I	Forward primer	AAGAGCCAGAGTGTCAGAATCT
	Reverse primer	AGCTCCAGTTGGTAATTTCTTGG
MAVS	Forward primer	GAATCCAGGTAGACGAAAGCC
	Reverse primer	GCCTACTACGGTACAGCATCAC
NF-κB	Forward primer	ATTCTGACCTTGCCTATCTAC
	Reverse primer	TCCAGTCTCCGAGTGAAG
GADPH	Forward primer	CTGAGCAAGAGAGGCCCTATCC
	Reverse primer	CTCCCTAGGCCCCTCCTGTT
FM1	Forward primer	GACCAATCCTGTCACCTCTGAC
	Reverse primer	AGGGCATTNTGGACAAAGCGTCTA

**Table 2:** Antibodies used for flow cytometry.

Antibody	Clone	Company	Labels Used
CD4	RM4-5	eBioscience	PE
IL-17A	eBio17B7	eBioscience	FTIC
CD25	PC61.5	eBioscience	PE-cyanine7
Foxp3	FJK-16 s	eBioscience	PE-cyanine5.5
IL-4	11b11	eBioscience	APC
IFN-gamma	XMG1.2	eBioscience	APC
